# Two pioneers of epigenetics: their different paths to chromatin research and DNA methylation, and general reflections on epigenetics

**DOI:** 10.3389/freae.2023.1334556

**Published:** 2024-01-17

**Authors:** Ute Deichmann

**Affiliations:** Jacques Loeb Centre for the History and Philosophy of the Life Sciences, Ben-Gurion University of the Negev, Be’er Sheva, Israel

**Keywords:** chromatin research, genomic causality, Adrian Bird, Gary Felsenfeld, reflections on epigenetics, insulator genes

## Abstract

The concept of chromatin as a complex of DNA (nuclein at the time) and proteins in the nucleus of eukaryotic cells was generated in the late 19th century. Since the late 20th century, research on DNA methylation that originated in the 1970s and chromatin research have also been labelled epigenetics, a term that originated in developmental biology in the 1940s. Epigenetics now comprises many different research strands related to the regulation of gene activity, such as chemical modifications of histones and DNA, chromatin organization, genome architecture, different types of RNA molecules, and others. To show the various paths on which epigenetic research has developed, I present research and reflections of two pioneers of what later became called epigenetics, Gary Felsenfeld and Adrian Bird. They began their scientific career in very different scientific contexts with both of them crucially contributing to the development of modern chromatin research and the understanding of DNA methylation, respectively. The article is based on authorized transcripts of interviews that I conducted with these researchers, focusing on those parts that are related to chromatin research and epigenetics as well as general reflections on epigenetics and biology.

## 1 Introduction

The concept of chromatin as a complex of DNA (then called nuclein) and proteins in the nucleus of eukaryotic cells emerged in the late 19th century and was the starting point for biochemical research on DNA and nuclear proteins. Interest in chromatin research declined at the beginning of the 20th century, among other things because of the rise of colloidal biochemistry with its focus on unspecific molecular aggregates, and the rapid development and success of classical genetics, which was based on genes as abstract entities (Deichmann, 2007; Deichmann, 2015). New research not only into the structure but also the function of chromatin began in the 1960s in the context of a new focus on the molecular biology of the eukaryotic cell and development.

Epigenetics was conceived by developmental biologist Conrad Waddington (1942) as the complex of developmental processes that lie between genotype and phenotype, and in which genes play a major role. These were Mendelian genes, that is, abstract factors, since the molecular nature of genes had not yet been elucidated. Research that pursued the questions Waddington raised was later mainly conducted under the term “developmental genetics.” In 1958, David Nanney suggested that control mechanisms of gene expression that could lead to different phenotypes of cells with the same genotypes and would be perpetuated during cell division be called epigenetic, though he, too, was unaware of their molecular basis (Nanney, 1958). His assumption that differentiated cells maintain their phenotype after several cell divisions was later included in the concept of cellular memory (Henikoff and Greally, 2016).

Cytosine methylation in DNA was likewise studied before the molecular nature of genes was known. In the 1920s, nucleic acids that were believed at the time to consist of small molecules of four nucleotides were isolated from *Mycobacterium tuberculosis* to identify its pathogenic determinant. One of the candidates was the nucleotide 5-methylcytosine (Mattei et al., 2022). In general, the idea of macromolecules became generally accepted only in the late 1930s, and DNA was demonstrated to be the material of genes only in the mid-1940s.

Since the 1970s, DNA methylation in higher organisms has been studied more extensively, also with regard to possible functions. But research labelled “epigenetics” remained marginal until the 1980s.

According to Bernhard Horsthemke (personal communication, 1 December 2023), some experimental studies in the 1980s on DNA methylation and cancer, imprinting, and X-chromosome inactivation (e.g., Mohandas et al., 1981; Feinberg and Vogelstein, 1983; Reik et al., 1987; Greger et al., 1989) contributed strongly to the explosion of modern epigenetics. With his speculation that methylated DNA might be transmitted not only through cell divisions but even across generations, Robin Holliday (1987) laid the foundation for a new, popular definition of epigenetics: At a meeting in 1996, epigenetics was defined as “the study of mitotically and meiotically heritable changes in gene function that cannot be explained by changes in DNA sequences” (Russo et al., 1996). This broad definition of epigenetics paved the way for the inclusion of chromatin modification, nucleosome positioning, prions and so on. The fact that research on chromatin modifications and DNA methylation has also been conducted under the label epigenetics marked the beginning of a molecular understanding of epigenetics that was no longer related to Waddington’s definition of epigenetics.

Today, the term epigenetics relates to all chromatin and DNA modifications and other transcription regulators that act in the context of chromatin. The diversity of researchers’ understandings and definitions of epigenetics increased dramatically. In molecular biology, cell biology, and chromatin research, epigenetics can relate to research on chromatin structure and function, nuclear organization, nucleosome remodeling, causes and functions of DNA and histone modifications, or the study of the self-perpetuation of signals as a requirement for cells to retain memories of past states. “Epigenetics” also refers to long non-coding RNA in transcriptional regulation and small interfering RNA as inhibitors of transcription and translation. Many epigenetic studies look at the interaction of DNA sequence-specific transcription factors, repressors, and RNA polymerases with histone proteins, chromatin compaction, looping, etc. in gene regulation processes. (For the history of chromatin research and epigenetics, see for example: Morange, 2002; Felsenfeld, 2014; Greally, 2018; Deichmann, 2015; Deichmann, 2016.)

In the following, I introduce the scientific biographies and research of two pioneers of what later became called epigenetics, Gary Felsenfeld and Adrian Bird. Starting their research in very different scientific contexts and with different goals, they crucially contributed to the development of modern chromatin research and the understanding of DNA methylation. Their work highlights the different origins of epigenetics and the diversity that has distinguished research on epigenetics since the late 20th century. For all the differences in their research, Felsenfeld and Bird agree that chromatin modification, DNA methylation, or other epigenetic factors closely interact with genetic or genomic factors. Felsenfeld made it clear that the first step in the control of gene expression is a transcription factor or other protein recognizing a particular DNA sequence, because this carries the information. “But once that happens, anything can happen.” (Interview with G. Felsenfeld by U. Deichmann, 17 December 2013; see below).

## 2 Gary Felsenfeld

Gary Felsenfeld received his BA at Harvard University in 1951 and PhD at Caltech in 1955. In 1961, he was appointed head of the Section on Physical Chemistry of the Laboratory of Molecular Biology of the National Institute of Diabetes and Digestive and Kidney Diseases, National Institutes of Health (NIH) in Bethesda, Maryland. He studied how chromatin proteins, including histones and regulatory proteins, and chromatin structure regulate gene expression and the processes of cellular differentiation and embryonic development. Later, he focused on the mechanisms that establish boundaries between large-scale chromatin domains within the nucleus and the role of regulatory proteins in genome organization. His group demonstrated that a particular protein (CTCF) is responsible for establishing an “insulator” activity to block selective interactions between enhancers and promoters and suggested that this activity might be due to its ability to establish loop domains within the nucleus. His group has also studied how changes in the chromatin-related regulatory mechanisms that govern cell growth and biochemical function impact diseases.

The following is an annotated and abridged excerpt of the interview that I conducted with Gary Felsenfeld on 17 December 2013, in his lab at NIH, Bethesda. It focuses on Felsenfeld’s transition from physical chemistry to biology and chromatin research and on his general thoughts about gene regulation. The interview highlights to what extent concepts of quantum mechanics and physical chemistry influenced biochemistry and illustrates how the transition from biocolIoidy to macromolecular chemistry impacted the emergence of molecular biology and modern research on chromatin and histones.

**FIGURE 1:**
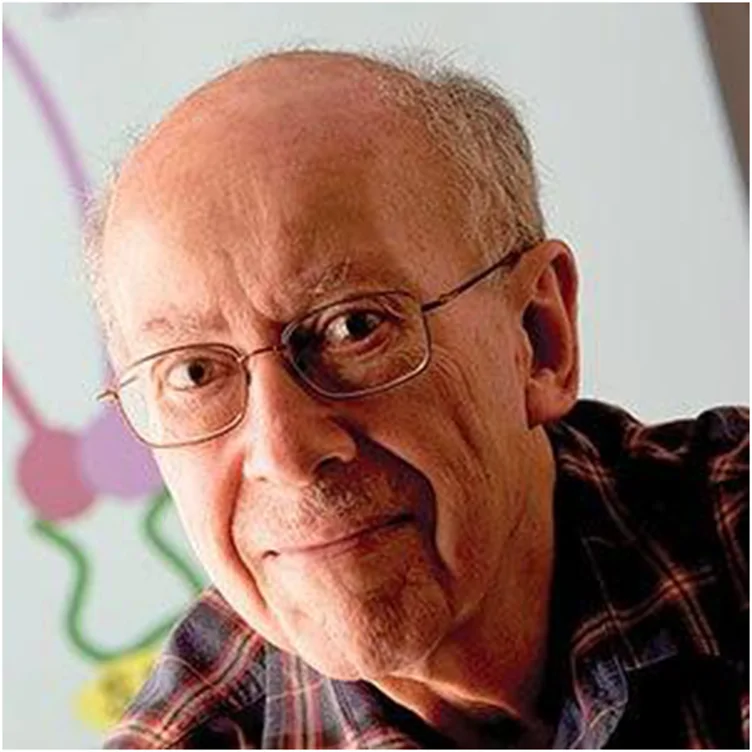
Gary Felsenfeld

### 2.1 From physical chemistry and quantum mechanics to the structure of synthetic polynucleotides

Felsenfeld studied biology and physics. One of his undergraduate tutors at Harvard University was the biochemist John Edsall, famous for his contributions to the chemistry and physical chemistry of proteins. Edsall, who was a friend of Robert Oppenheimer’s, introduced Felsenfeld to quantum mechanics. Felsenfeld remembered: “[Edsall] applied physical methods to biological problems, particularly the properties of proteins in solution. I was interested in the biology, and I had fairly strong quantitative inclinations. And he said the biology of the future will involve physical chemistry. The term ‘molecular biology’ did not yet exist [in the 1940s]. The idea would have been thought ridiculous. So, I did an undergraduate thesis with him, and in my last year when we discussed what I should do next, he said that I should go out to Caltech, which was the center for the study of this kind of biology–to the extent that there was. There was crystallography and there was a strong emphasis on physical chemistry, so that’s what I did. Edsall was probably the first biochemist to study quantum mechanics. He told me–and he’s written this as well–that he first learned about Schrödinger’s formulation of quantum mechanics from J. Robert Oppenheimer.” Felsenfeld began to study quantum mechanics under the tutelage of Edsall when he was still an undergraduate. In 1951 Linus Pauling gave a lecture about the alpha helix at MIT. “You can’t imagine how amazing that was.” At the time, “the structure of proteins was guessed at. And they taught us something called the cyclol structure proposed by Dorothy Wrinch. We had to learn that, but of course, it was totally wrong.” (In the 1930s, mathematician Dorothy Wrinch proposed a structural model of globular proteins, in which amino acids spontaneously interact to create cyclol molecules, a model that was later overwhelmingly refuted.)

It was his knowledge in quantum mechanics that led Pauling to solve the structure of the alpha helix: “You know that the reason that Pauling came upon the alpha-helix, and others missed it, is because they did not appreciate that all the atoms around a peptide bond have to lie on a plane. Pauling knew that two ways. He knew it, first, because his understanding of valence bond theory and the behavior of chemical bonds allowed him to predict that this would have to be the case. Secondly, and more importantly, in pursuit of that, he had arranged for [Elias J.] Corey and others who were associated with him to solve the crystal structures of di-peptides and things like that, which proved that the peptide bonds were co-planar. And therefore, when he built his models, he was left with a very small subset of the otherwise rather long list of possible structures of a polypeptide.”

In 1951, at Caltech, Pauling became Felsenfeld’s thesis advisor. Felsenfeld’s thesis was on the theory of ferromagnetism. This had nothing to do with biology, but he nevertheless thought that “this was going to be a preparation for a career in biology because the biology of the future would make use of these kinds of techniques.” At some point he was wondering whether he could make a career in quantum mechanics and went to see Richard Feynman, whom he knew. Feynman asked him: “Well, do you see any simplification in the field of quantum chemistry–anything new that would simplify this complicated subject?” “I said that I didn’t think so. Then he said, ‘Well then, get out.’, which is what I wanted to hear anyway.”

Felsenfeld then studied theoretical physical chemistry with biology as a minor. He remembered Francis Crick and James Watson coming to Caltech in the early 1950s to a big meeting on proteins that was convened by Pauling. At that time, Pauling also announced that he would describe the structure of DNA. Felsenfeld had no idea why DNA should be important: “It wasn’t discussed much in my courses. They talked about thymus nucleic acid and yeast nucleic acid - one was DNA and the other, RNA. And of course, coming out of the dark ages of polymer chemistry in the early 1930s, before then, polymers were all thought to be loose aggregates of small molecules. For quite a long time, the nucleic acids were thought to be aggregates of fundamental sub-units consisting of the four different nucleotides. And, in fact, the recipe for studying RNA at one point was to boil it in sodium hydroxide to disrupt the non-covalent bonds and get the essential tetrad of four nucleotides–of course that’s how you degrade RNA: you hydrolyze the bonds. And similar ideas existed about DNA.”

Felsenfeld here relates to the colloidal concept in biochemistry that became the dominant view in the chemistry of substances and processes in the cell since around 1900. According to this concept, all biologically relevant substances like proteins and nucleic acids were biologically active colloidal aggregates of undetermined composition. Evidence for the macromolecular nature of proteins and DNA was presented since the late 1920s but was not immediately generally accepted. The concept of macromolecules was a prerequisite for the study of the molecular basis of biological structure and function, particularly of proteins and DNA that is molecular biology (see Deichmann, 2007).

After his graduation, Felsenfeld wanted to go to Copenhagen to study protein chemistry. But Pauling wanted him to have further training in quantum mechanics even though Felsenfeld intended to do biology, and he sent him to the lab of Charles Coulson in Oxford, Coulson being a mathematician who was renowned for applying quantum mechanics to molecular structure - at the time graduate students could not decide what to do on their own. A year later, in 1956, Felsenfeld returned to the US, where biophysicist Alexander Rich invited him to join the NIH as an officer in the Public Health Service—though the Korean war was over, Felsenfeld had to serve his military time, and the work in the Public Health Office satisfied this requirement.

At that time, Severo Ochoa and Marianne Grunberg-Manago were synthesizing the first polynucleotides, and Rich, who knew Ochoa, received samples of his polyadenylic and polyuridylic acids. Together with Rich and David Davies, Felsenfeld began working with synthetic polynucleotides. He succeeded in creating a triplex structure and began to study what stabilized these negatively charged polynucleotides (Felsenfeld et al., 1957). He continued this work when he joined the biophysics department at the University of Pittsburgh.

### 2.2 The beginning of work on histones, chromatin, and DNA-specific regulatory proteins

“So, I began to study how ionic conditions affected the stability not only of these synthetic structures, but also of real DNA. From there, the next thing was to use larger and larger positively charged ions and I did that. I studied polylysine and polyarginine, both peptides carrying positively charged amino acids, and things like that as models of what might be going on in the nucleus. Because we already knew there were histones in the nucleus and they were positively charged, with lots of lysines and arginines.” He stopped working on synthetic polypeptides and began to purify and use histones, the beginning of his interest in chromatin. He remembered:

“I began to be interested in the actual structure of chromatin and how the histones covered the DNA, and we developed nuclease probes to look at the chromatin structure. And then we hit on the chicken beta globin locus, as a perfect test system. We were physical chemists, and you have to know that making chromatin in those days was more or less like Macbeth and the Witches Brew. People made chromatin from calf thymus; this was the standard way to make chromatin. The trouble was that this tissue is full of nucleases and proteases, and you had, perhaps, three or four hours—it took a day to make it, and at the end of the day you started the experiment. You couldn’t let it sit overnight. And you never knew what you were getting.”

“At this point, I think it was Harold Weintraub who for other reasons was beginning to study chromatin in chicken erythrocytes. Weintraub was interested in the chicken beta globin locus as a model locus for studying gene expression. Chicken erythrocytes keep their nuclei—birds keep their nuclei whereas we do not. And the nuclei are shutting down, really. But they still contain a lot of the active components, and the main thing is that they’re almost free of nucleases and proteases. So, it’s dead simple—you get blood from a chicken, you break open the cells, you centrifuge and wash a couple of times, you have a beautiful preparation of white nuclei, and then you can do what you want. So, you can study the chromatin of an active gene in a cell where you can hope for relative stability and resistance to degradation. Perfect for a physical chemist.”

Felsenfeld and his colleagues were able to treat the blood cells in such a way that they could float DNA and nucleases into them at a time when transfection, the artificial insertion of nucleic acids into cells, was very difficult in eukaryotes. He remembered that the procedure was “just perfect–again for a physical chemist–it required no sophistication whatever. It worked every time, it was reproducible.” Their interest in regulation led to the identification of the first of the GATA proteins, GATA1 (Evans and Felsenfeld, 1989). (GATA proteins are transcription factors that bind to a DNA sequence motif found in the cis-regulatory regions of many hematopoietic genes): “We discovered it at the same time as Stewart Orkin at Harvard. And that turned out to be the founder of a big family of GATA proteins that are major regulatory proteins. GATA1 is very important in erythroid cell development. This was at a time when MyoD was being discovered [the MyoD or myoblast determination protein plays an important role in the regulation of muscle differentiation]. People were beginning to realize that there were cell-type-specific, developmentally specific regulatory proteins.”

The basic subunit of chromatin is the nucleosome.

“The nucleosome was first discovered and characterized structurally and chemically by [Roger D.] Kornberg and [J. O.] Thomas and identified in the microscope by [Don and Ada] Olins, who actually had a cover on the front page of Science–showing this bead-on-a-string structure. Before that there were a lot of uncertainties about the histones, because there seemed to be a lot of them, but it turned out that many of them were just degradation products. There were really only five altogether. Four that formed this central complex called the nucleosome core, and one on the outside. The core contained an octamer of histones (two each of the four central kinds). Around this octamer, two super-helical turns of DNA are wrapped. About 165 base-pairs of DNA, locked in place by the fifth histone. And that is the first order of compaction. Because part of the purpose of chromatin is to compact the DNA. So, you get this bead-on-a-string structure, and that’s what has been most studied. But it folds up further into far higher orders of compaction, and we studied the next order of compaction. That is still being actively studied, and it is still a matter of uncertainty. We don’t do much of that anymore.”

I asked whether there is a relationship between the structural units and their biological function. He answered:

“If you had asked me five years ago, the answer would have been much more certain. The thought was that these higher orders of structure were ways to compact the DNA that was not needed for function in a particular kind of cell. And that the structure was opened for transcription. And that may still be true. But at the level of the nucleosomes themselves, it is clear chromatin plays a major role. People began to ask whether the histones had to be removed to accommodate transcription, or how chromatin structure had to be modified. At about that time Vincent Allfrey at the Rockefeller Institute began to provide evidence that histones associated with transcriptionally active chromatin were modified, particularly by acetylation of lysines.”

“I think most people paid too little attention to this. This was a mistake, and Allfrey deserves a lot of credit for persevering. But what really made the difference was that people working in *Tetrahymena* [a unicellular eukaryote] and yeast began to isolate enzymes that modified histones or affected chromatin structure. So, the problem always was, with Allfrey’s results, chromatin containing active genes had acetylated histones, but was that just an accidental correlation? But with yeast genetics it was possible to show that mutating the enzymes responsible for histone modifications such as acetylation had a phenotype, a direct effect on yeast growth. Two or three groups really were able to show that. David Allis identified an enzyme in *Tetrahymena* that acetylated histones and realized that it was similar to a protein in yeast, and it was known that when the gene for that protein was mutated in yeast there were effects on growth. That made the connection between the biology and the chemistry. Stuart Schreiber discovered an enzyme in mammalian cells that removed histone acetylation marks and was related to a yeast gene known to regulate transcription. And then there was the discovery of enzymes that move the nucleosomes around on DNA, which also had significant phenotypes detected through yeast genetics. That was a different class of proteins—chromatin remodeling enzymes.”

### 2.3 The complicated mechanisms of the regulation of gene activation; the problematic term of epigenetics

Felsenfeld thinks that contrary to the idea of a histone code, where histone marks could be associated with active or inactive genes, “gene activation is not so simple. And, in fact, you have to do it almost individual gene by gene. Each has its own way of activating, and each will have its own pattern of events leading to activation. In some cases, you completely mask the promoter with a nucleosome, and that tends to make it difficult for a regulatory protein to get in without help from histone modifying and remodeling enzymes. In others, you let transcription initiation occur, the RNA polymerase gets on board, but then is paused and stuck, and then until you do some modification later, perhaps of a histone, it will not go.”

“So, there are modifications that alter initiation, binding of RNA polymerase, and there are modifications that alter elongation. And there are modifications that affect splicing - our RNAs are spliced if they have introns. And many are alternatively spliced. There are many genes with multiple introns, and the actual final RNA product will not use all of them, but a subset. And under different circumstances, a different subset. So, you get different proteins out of the same coding region. And that also is controlled in part by these epigenetic marks. But this, of course, doesn’t address the question that I addressed in my review, which is whether this is truly epigenetic or really counts as just a part of the biochemistry—although a very complicated part and an essential part. The rather loose use of this term has roused such animosity among the people who think that this is just a fashionable term to use.”

In his 2014 review article, Felsenfeld made it clear that most of the modifications relevant for gene activation are not epigenetic in the sense that they are inherited. In the interview, he explained: “they are not marks that are transmitted through cell division or the germ line.” To him, the term “doesn’t make any difference from the point of view of the science because whatever you call these things, they are the mechanism for the regulation of gene expression, and that’s what you have to study. So, you can call it anything you like. It is too bad it got called epigenetics. Because this implies something about inheritance, and the role of the environment, and we are still a long way from understanding that.”

It has not yet been clearly found out how these mechanisms are controlled: “They are controlled in as many different ways as nature can accidentally devise. One of the things we’re finding out is that in fact nature has tried everything. Almost anything you can imagine in the way of a mechanism is beginning to turn up. Because evolution is, as I always say, not constrained to–not being told to write a textbook in which 50 pages will be devoted to epigenetics, and we better get it all in there. It keeps writing new pages. And there are no rules–except the rules of chemistry and physics.”

“So, as I said in the review, the first thing that has to happen in the control of gene expression is that you have to have a transcription factor that recognizes a DNA sequence. In the promoter, usually. Sometimes an enhancer, but it has to be an interaction between a protein that actually recognizes a particular, and usually unique, DNA sequence. That is the initial step. That carries the information. But once that happens, anything can happen. The next thing may be that you recruit a nucleosome remodeling enzyme for the nucleosome that is next door, which pushes that nucleosome out, and now the next factor can arrive, and then that factor may further recruit histone-modifying enzymes. That loosens the chromatin structure, perhaps. And finally, the RNA polymerase binds and will recruit further histone modifying enzymes. So, as it moves along the gene, transcribing it will modify the nucleosomes—maybe loosen them up so that some of the histones can jump off and then back on again—not necessarily the whole nucleosome, just a subset of the histones. But each gene will be different, and the order of events could be different under different circumstances or different kinds of cells.”

### 2.4 Nuclear organization and the role of regulatory proteins in it

Many years later, the large-scale chromatin organization and the boundaries between independently regulated domains became a focus of Felsenfeld and his collaborators. They studied boundary elements that prevent the spread of condensed chromatin into transcriptionally active regions as well as those that generate ‘loop’ domains. These domains either bring together distant DNA sequence elements or segregate them into separate loops. This research was connected to his earlier work on the beta globin gene in chickens:

“One of the things that we’re interested in now is connected to our previous study of the chicken beta globin locus. Very early on we had mapped the histone modifications over the locus and made one of the early identifications in a vertebrate of histone modification *versus* activity. We noticed that this entire beta globin locus, which contains all four beta globin genes that are developmentally expressed either in the embryo or adult, was embedded in a whole bunch of silent chromatin. The question was, why didn’t the silent chromatin spread and swallow up the active, and why didn’t strong enhancers located nearby turn on the globin genes inappropriately?”

“So, we began to look for boundary elements, something that would keep the wolves away. We found that there was a region just at the edge of the beta globin locus that contained some proteins that blocked the advance of silent so-called heterochromatin. And we also found a protein called CTCF [a zinc-finger protein] which, when bound to DNA, keeps enhancers that are outside, or any other activating signal that is outside, from getting in. CTCF was a known protein but had not been identified as having that function. This kind of protein is called an insulator.” (Bell et al., 1999)

“It became a major object of study over the last 10 years for a large number of scientists. And we are included. It turns out that if you have a CTCF bound to DNA here and another bound at a distant site, they can find each other over long distances. They form a loop. And indeed, if your gene is inside the loop and your enhancer is outside, it will keep the enhancer from reaching the gene, which will tend to be silenced. But there are other situations in which the enhancer and promoter are both inside the same loop and then it tends to bring them together, and the gene may be activated. And so, it turns out that CTCF is the foundation of a lot of large-scale nuclear organization that is designed to help regulate long-distance interactions in the nucleus.”

“So now, lots of people are studying long-range interactions in the nucleus and how they affect gene expression. This is made possible largely because of a discovery some years ago by Job Dekker of a method for mapping physical contacts within the nucleus. This nuclear architecture is essentially established right after cell division. Very recent studies by Dekker and his collaborators show that the structure is disrupted and a new one established in mitotic chromosomes. But once you get through cell division, then the whole genome is in loop domains—on average, perhaps a megabase in size. And in addition, genes with related functions, even on different chromosomes, will often tend to bunch together. … The net effect is that genes that require a specific factor will find it concentrated at the appropriate cluster. But I think it is still not clear whether the concentration is a cause or an effect. That’s what people are interested in now. That’s the level at which all of this is now being explored.”

### 2.5 Failures and dead ends

At the end, I asked Felsenfeld whether he had encountered scientific failures or dead ends in his own or other people’s work on chromatin and histones. He answered: “Actually, we didn’t have many. Except we did not see the nuclear sub-structure, I have to say. We were not thinking that way. I was thinking in terms of the biology and not the structure at that point. It was a mistake. You have to think about structure before biology.”

“People tried to study the properties of the individual histones. They even solved crystal structures. And these turned out to be of no interest whatever. Because those histones never—in vivo—ever exist, except as part of a complex with other histones or with chaperones.”

“And there were a lot of experiments trying to transcribe chromatin *in-vitro*, isolate chromatin and then show that the transcript you got was restricted, as you might expect. We did some like that, and I think ours were OK. But a lot of it was actually looking at endogenous RNA, which was there because you couldn’t purify it away. RNA was actually part of the original chromatin rather than something that was created when you tried to transcribe.”

To summarize this part: Gary Felsenfeld’s scientific biography illustrates a physical chemist’s path to research now called epigenetics, who began his work before the structure and function of DNA were elucidated. Starting with work on the structure of synthetic polynucleotides, he succeeded in groundbreaking research in the areas of histones, chromatin, and DNA-specific regulatory proteins. Felsenfeld emphasized the importance of studying structure, particularly the substructure of the cell nucleus and the organization of the genome. Still an active researcher, his work spans more than 7 decades. Unlike Felsenfeld, Adrian Bird began his research when the structure and function of DNA were known. As the next section shows, he was a molecular biologist when he began to work on the questions of DNA methylation and chromatin structure.

## 3 Adrian Bird

Adrian Bird is a molecular biologist focusing on the biology of the genome and genomic regulation. He graduated in biochemistry from the University of Sussex and obtained his PhD at Edinburgh University in 1970. Following postdoctoral experience at Yale University and the University of Zurich, he joined the Medical Research Council’s Mammalian Genome Unit in Edinburgh. In 1987 he moved to Vienna to become a Senior Scientist at the Institute for Molecular Pathology. Since 1990, he holds the Buchanan Chair of Genetics at the University of Edinburgh.

Bird and his working group identified CpG islands in the vertebrate genome, that is, genomic DNA that is full of CpG sequences that are not methylated and that became understood to be near promoters. He discovered proteins that read the DNA methylation signal to influence chromatin structure. Mutations in one of these proteins, MeCP2, cause the neurological disorder Rett syndrome, and he discovered that the resulting severe neurological phenotype is reversible, at least in mice.

The following is a shortened and annotated excerpt of the interview that I conducted with Adrian Bird on 28 May 2018, in his lab at the University of Edinburgh. The focus is on Bird’s early research in molecular biology that led him to the study of DNA methylation, his work on CpG islands, and his general reflections on biology and epigenetics.

**FIGURE 2:**
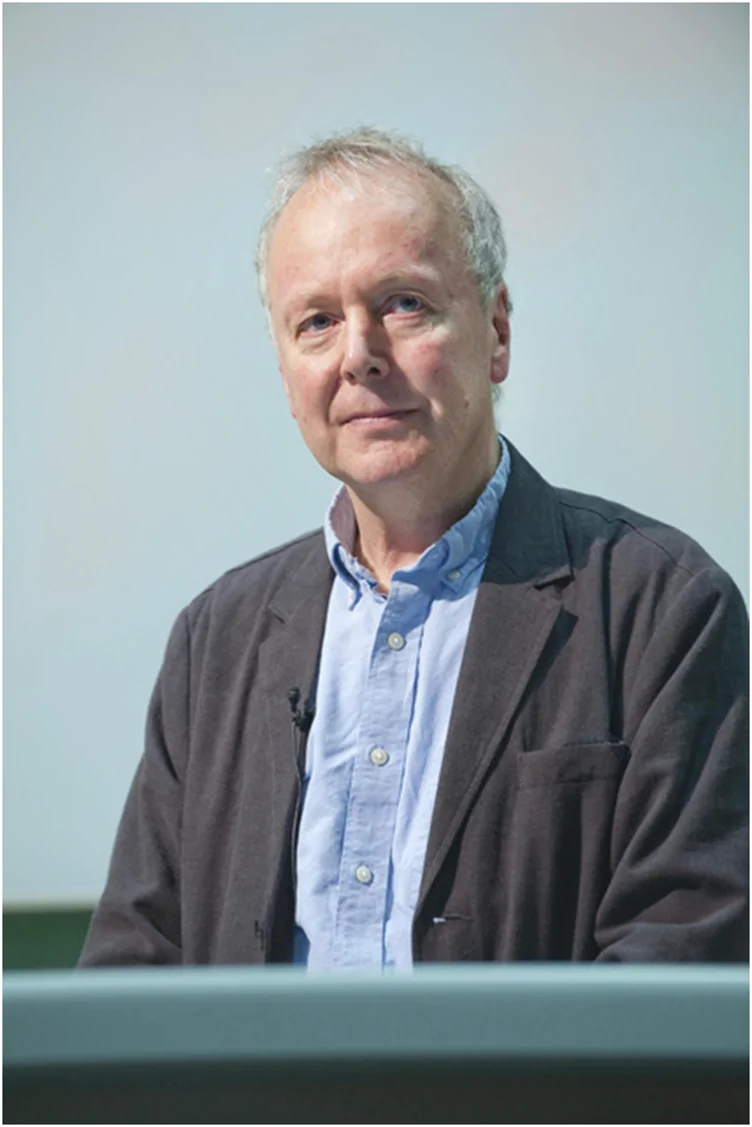
Adrian Bird

### 3.1 From gene amplification to DNA methylation

In the 1960s, Adrian Bird worked on gene amplification in the frog *Xenopus laevis* for his PhD. Don Brown and Joe Gall in the United States had shown that in frogs the oocytes amplified their ribosomal RNA genes, taking them out of the chromosome and making thousands of copies. Bird told me that gene amplification at the time seemed like a paradigm for the way development might work. But amplification turned out to be an exception, not a paradigm, and a few years later it was shown that the genome does not change much during development. The Don Brown lab also showed that the amplified ribosomal RNA genes differ from the normal chromosomal copies with respect to DNA methylation - “there is lots of CpG methylation in chromosomal DNA, but the amplified ribosomal DNA is completely free of 5-methylcytosine (5 mC)”. (CpG is the abbreviation of Cytosine—phosphate—Guanine sequence in DNA).

“Knowing about this difference, I was able to interpret an experiment I did in Zurich when I was in Max Birnstiel’s lab. Restriction enzymes as a way of mapping DNA had just come in, and Hamilton Smith, who won the Nobel Prize for finding that restriction enzymes could be used for mapping, appeared in Zurich for a sabbatical. The first thing he did was to make a restriction enzyme, so I tried it out on some samples of ribosomal genes that I had in the fridge. When I compared amplified with chromosomal ribosomal RNA genes after cutting with this restriction enzyme, the patterns turned out to be completely different even though they should have the same DNA sequence. The chromosomal stuff didn’t get cut and the amplified stuff got minced into tiny little pieces. The explanation, which took a while to sink in, was that the difference was due to DNA methylation. The enzyme cut at a site with a CpG in it and methylation of the C stops the enzyme from cutting.”

“Because the difference in cutting pattern was due to DNA methylation, this result meant that one could use this restriction enzyme to map methylated sites. To demonstrate this, we compared the cleavage map for the methylated chromosomal genes, which told you which sites were blocked, with the map when no methylation was present, which told you where all the CpG sites actually were. That gave us the first map of methylated sites in the genome (Bird and Southern, 1978). Before that, one knew that there was methylation in the genome, but not where it was. So, this was a breakthrough in the field.”

### 3.2 The discovery of CpG islands

The discovery of CpG islands, that is, clusters of the dinucleotide CpG, was the result of a comparison Bird and his group conducted of methylated and unmethylated sections of DNA in different organisms. They carried out what he called a “phylogenetic exploration”: They used restriction enzymes, which frequently cut in genomic DNA, on various organisms. They picked up marine organisms from the local marine biology stations in St. Andrews and Millport on the west coast of Scotland and found that “in most cases the DNA was quite well cut, suggesting widespread absence of genome methylation. But for vertebrate DNAs–frogs, birds, fish, mammals–the enzymes hardly cut at all; there was so much DNA methylation that all the sites were blocked.”

The group had the idea of end-labelling the fragments to detect trace amounts of unmethylated DNA, work that was conducted by Bird’s graduate student David Cooper. Instead of seeing the *weight* of DNA by gel electrophoresis, they saw the *number* of fragments. “Suddenly he saw a big blob at the bottom of the gel that we hadn’t been able to see before. It just looked like an artifact, really. But we pursued it, and it turned out that this was derived from GC-rich non-methylated domains that were really quite short. We cloned those sequences and showed that they were derived from the promoters of genes. Based on the rather minimal sampling of the genome, we had detected the CpG islands.”

Bird added that this was an idea whose time had come; it was in the air, but it had not yet been generally accepted. The results of his group had really shown that there was a category of genomic DNA that was full of unmethylated CpGs and that were located near promoters. “Vertebrates have just a tiny fraction of unmethylated genome, and it is in the CpG islands.”

His group later showed that most vertebrate CpG islands (CGIs) are sites of transcription initiation and that “shared DNA sequence features adapt CGIs for promoter function by destabilizing nucleosomes and attracting proteins that create a transcriptionally permissive chromatin state. Silencing of CGI promoters is achieved through dense CpG methylation or polycomb recruitment, again using their distinctive DNA sequence composition” (Bird et al., 1985; a later review is in Deaton and Bird, 2011).

### 3.3 Particularities of CpG islands; the mutagenic effect of DNA methylation

I asked Bird if it was possible to distinguish these CpG islands clearly from other parts of the chromosome. In his answer, he also highlighted the highly interesting and often overlooked fact that DNA methylation is mutagenic:

“[CpG islands] are dramatically different. First of all, in terms of methylation, the paradox is that there’s no DNA methylation there despite a 10-times higher density of methylatable sites (i.e., CpGs) compared to the rest of the genome. There are two reasons. The first—the base composition of the DNA of the CpG islands is rich in G and C. Most of the genome is 40% G plus C, which is the same as 60% A plus T, but in CpG islands it’s the other way around. In fact, it’s more extreme: 65% G plus C, so by chance alone you end up with more CpGs there. But that’s not the biggest reason. The biggest reason is that methylation is mutagenic. You might not have heard that before. One of the most important biological, biomedical attributes of cytosine methylation in DNA is that it causes mutations. The reason is that cytosine is prone to deamination, and loss of the amino group turns cytosine into thymine.”

“It’s a fascinating piece of biology. Water causes cytosine to deaminate so that you get uracil—it happens about 100 times per cell per day. Uracil is a natural base in RNA, but in DNA it would be a serious source of mutations. What has happened during evolution is that genomes have methylated the uracil so that now they can distinguish the deamination product of cytosine from a normal DNA base. So now when you deaminate methylated cytosine—it becomes a big problem, because instead of uracil, you get thymine. There is a whole machinery for removing uracil from DNA—repairing it. But it will not touch thymine. The repair mechanisms for this change, though there are some, are very inefficient and, as a result, about a third of all the point mutations that give rise to human genetic diseases are at CpG. It’s one of the most important biological features of DNA methylation. People tend to skate over it, but I think it’s absolutely fascinating!”

The mutagenic effect of cytosine methylation (mC) was first shown in bacteria by Coulondre et al. (1978). Subsequently, Bird (1980) demonstrated that this effect is responsible for the well-established deficiency of the dinucleotide CpG in heavily methylated vertebrate genomes and thus that mC mutability applies in eukaryotes. He believes that the fact that evolution has kept for so long a mechanism that has mutagenic effects, has to do with the cost-benefit component of evolution, assuming that the benefit was bigger than the cost.

“Yes, we’ve smeared a mutagen all over our genes, basically. It’s a relatively mild mutagen; the mutation rate for methyl cytosine is approximately 10 times higher than the mutation rate for any other base. Note that CpG islands have not been methylated for millions of years so they have not lost their CpGs. And that’s the second reason why there are so many CpGs there.”

### 3.4 Gene regulation, the environment, and heritability

Bird emphasized that “genes are regulated by proteins which usually have nothing to do with DNA methylation. They are sequence-specific DNA binding proteins or transcription factors. They are, if you like, the ‘smart’ molecules in the cell as they can tell one bit of DNA from another by reading the base sequence. After all, that’s the main thing that distinguishes one bit of the genome from the other. DNA methylation is not involved in short-term regulation of genes.”

In several publications, he expressed the view that there is an intricate interaction between sequence-specific binding proteins and chromatin structure. He told me: “I think if you ask people about epigenetics, the first thing they talk about is the environment impacting on the way genes are expressed. And with epigenetics you also have the concept of heritability, which offers a way in which you could get environmental information put into the genome and then transmit it. But, in fact, the evidence suggests that the genome is heavily insulated from the environment, actually. It is not, sort of, waiting like a young bird in a nest for environment input. To me, it seems that for a lot of aspects of genome management, like DNA methylation, the logic is internal to the cell. It is not dependent on the environment to give the instruction. And even in plants, where the long-term impact of the environment on gene regulation is best characterized, epigenetic changes are not adaptive, but seem to be random. The environment is not informing the genome. If the logic is internal, then the DNA sequence is likely to be impacting on the epigenome. And there’s quite a lot of evidence that’s the case.”

Bird deplored that the beliefs of the environment directly impacting the epigenome, dominating the genes, and effects being inherited, are meanwhile widespread and even appear in school curricula. He remembered a questionnaire from a Swedish group “asking how I thought epigenetics should be incorporated into the teaching of psychiatrists and psychologists and sociologists and school pupils, and I found it extraordinary; it was basically inviting an attack on the teaching of genetics. It was all yes/no, agree/disagree, but the questions were formulated in such a way that clearly, they gave you the option of saying that genetics was vastly overrated and the environmental influence on the epigenome underlay all sorts of phenomena that had previously been considered to be relatively hard-wired. The way the argument is often presented is that people who believe in genetics are old-fashioned and not moving with the times. They are reactionaries and this new epigenetic revolution promises to liberate people from their genomes.”

Bird also does not believe that cellular memory is caused by DNA methylation: “*Drosophila* doesn’t have any methylation, nor does *C. elegans*. To me, cellular memory is not as remarkable–it doesn’t beg for an explanation–because when a cell divides, it’s got its transcription factors there, and then it splits in half and afterwards each daughter cell has still got its transcription factors. So, any positive feedback loop can re-establish the cell state. It is not a miracle that when something divides in half and both halves have the same constituents, that both continue to be the way they were before.” (See also Bird, 2002.)

He added that the cellular memory conferred by DNA methylation would be rather imperfect memory because it is error-prone and cannot be sustained after a few cell divisions: “So, you may have to reinforce continuously–in which case it is not sufficient by itself to memorize cell state. But secondly, except in a few cases of transcriptional shutdown, it doesn’t look as though most gene expression programs are remembered by DNA methylation at all. On top of that, DNA methylation doesn’t seem to be the key component that regulates gene expression, as we were saying before. So, if it is not critically involved in regulating gene expression, then what is being remembered?”

Even though there are copying mechanisms that help keep the methylation on the DNA during mitosis, Bird thinks that there is some evidence that silencing has to be continuously reinforced: “In other words, it is not the cell says, ‘methylate this’ and can then forget about it; if you forget about it, it may lose its methylation gradually.”

### 3.5 Organisms as “cumbersome bureaucracies”

Bird drew attention to the fact that switches in the liquid state of a cell cannot be easily brought about and that either a high density of methylation or the cooperation of many factors are needed to silence or activate genes:

“When a CpG island gets methylated, you have very dense methylation and that then shuts down that gene. That is what happens on the inactive X, it’s what happens on certain imprinted genes. But for globin genes and growth hormone and keratin and genes like that, they don’t have CpG island promotors. And quite a lot of tissue-restricted, high expression genes characteristic of terminal differentiation states don’t have CpG islands either. In these cases, the methylation density is low, and its influence on gene expression has been almost impossible to show convincingly. I would argue that DNA methylation density is an important parameter - if you don’t have a high density, then the repressive effect of methylation is terribly weak.”

He also makes it clear that DNA methylation is not associated with all genes that are turned off and that genes have to be shut down by many factors, such as polycomb, absence of activators, and a lot of sequence-specific repressors: “DNA methylation is hardly ever found to be responsible for shutting down tissue-restricted genes to my knowledge. And in CpG island genes, methylation is absent the whole time. Only on the inactive X, imprinted genes and a few other cases do you get reproducible methylation of CpG islands that clearly contributes to silencing.”

He uses X chromosome inactivation as an example to show that none of these epigenetic mechanisms is enough. In most mammals, one of the two X chromosomes is randomly and permanently silenced in females in all cells other than egg cells. This ensures that females, like males, have only one functional copy of the X chromosome in each body cell: “There you have methylation of the DNA, you have late replication during S-phase, you have polycomb, which is an independent repression mechanism, you have histone deacetylation, position in the nucleus, and all of these things matter. I think it is because making a switch in a liquid state is not very easy. We tend to think of cells as analogous to computers. But computers work with switches that are binary: 1 or 0. In liquid, however, when you’ve got chemical reactions going on, nothing is ever 0.”

“This means that cells have to indulge in tricks to make switches. And one of the ways they do it is, rather than spend huge amounts of effort making a perfect switch, they stack imperfect mechanisms one upon the other. So, let’s say you have a mechanism that is 90% efficient that silences a gene. That means that 10% of the time the gene is going to switch on by mistake. Now let’s say you have an independent mechanism operating on the same process which is also 90% reliable; you have achieved 99% efficient silencing. If you have three such separate mechanisms, 99.9%, etc. So, by stacking inefficient mechanisms one on top of the other, you can get really, really good repression. DNA methylation is one of those mechanisms, but it’s not all of them.”

“I actually increasingly think of organisms as rather cumbersome bureaucracies! They are like human bureaucracies. Bureaucracies have a bad reputation, but actually they are jolly stable! Bureaucracies can be inefficient for ages and still continue, so biologically that’s a good property!”

### 3.6 DNA methylation patterns are evenly distributed over the genome

“If you look at the screenshot of where the methylation is in the genome, it’s rather evenly distributed. CpGs have a probability of about 70% of being methylated, except for the CpG islands where methylation disappears. In a liver or in blood or skin or brain, the pattern looks remarkably similar. So, a striking feature of methylation is its constancy. But because we are more interested in differences than similarities, you don’t get any plaudits for pointing out things that are the same. As a result, an outsider gets the impression that the DNA methylation patterns in different tissues vary enormously. But they don’t!”

He explained that some of the differences may be important. An example is the drop in DNA methylation over genes that are highly expressed in the brain. But he believes that “one should always point out that the differences are against a background that is strikingly constant on average. Otherwise, you get the view, and you still find it in the literature after many years, that DNA methylation switches gene expression patterns and I just don’t think the evidence supports that.”

He demonstrated the fact that the gene activities between different regions are more different from each other than the methylation, in T cells: “When you are challenged by some toxin or bacterium, then T helper cells turn into Th1 or Th2 [two classes of T helper cells that play an important role in the immune system] depending on what sort of battle they’ve got to fight. You can get these from mice and turn them into Th1 or Th2 in a dish. There are thousands of gene expression differences between Th1, Th2, and the T helper cells, and almost no DNA methylation differences. We were astonished by that, but others have seen the same. For example, muscle development, taking myoblasts and fusing cells to make a multi-cellular syncytium where all the nuclei are in one cytoplasm going on to make actin-myosin striations–virtually no changes in DNA methylation. The conclusion is that gene expression is not being regulated by DNA methylation, but so ingrained is the idea that it is, that it’s almost impossible to displace.”

### 3.7 The inextricable link between genetics and epigenetics

In many of his papers Bird expressed the view that epigenetics and genetics are closely linked (see, for example, Bird, 2002; Bird, 2013). He explained this connection using the example of two neurological disorders, the causes of which he had studied for many years:

“The two examples of Rett syndrome and Fragile X syndrome are cases where people refer to them sometimes as epigenetic diseases because they both involve DNA methylation. Rett syndrome involves a reader of the methylated sites and Fragile X syndrome involves massive methylation of a promotor region of a gene which leads to its shutdown. In that sense they are epigenetic. But they both, as I stressed before, are caused by mutations that change the DNA sequence. The primary change is the DNA sequence, and the secondary consequences are the epigenetic changes. In the case of CpG islands, it’s pretty clear that the chromatin structure, which I haven’t mentioned before, is different there. There is, for example, methylation of histone H3 lysine 4. This is characteristic of CpG islands, and this is recruited by a protein that binds to non-methylated CpG sites, of which there are a lot in CpG islands - and very few elsewhere. This protein is going to CpG islands because of the DNA sequence; nothing to do with epigenetics. The protein is going there and recruiting the enzyme that methylates the histone. Histones are methylated, acetylated, ubiquinated, phosphorylated—they have lots and lots of chemical moieties on them, many of which are not terribly well understood.”

“Coming back to Cfp1 [CpG binding protein], the DNA sequence at CpG islands is informing the structure of the epigenome by directing methylation to histones. So, I see the epigenome as primarily the client of the DNA sequence. And if the epigenome changes, it’s because developmental programmes put different mediator proteins in place that are adapting the epigenome based on the underlying DNA sequence. This is very different from the view that the environment is dictating the epigenome.”

I end with a quotation from Bird (2013), where he reminds us that “Sixty years after the double helix, the intellectual excitement of the golden age of biology deserves more than ever to be shared with all comers, but it should be borne in mind that, in biology, ideas are relatively cheap. It is their rigorous testing that takes time and ingenuity. Until then, an ever-present danger is that views gain credence because they fit with preconceived notions of what feels right. Transgenerational epigenetic inheritance, for example, opposes the notion—unpalatable to some—that many human attributes are genetically ‘hard-wired.’ To counteract wishful thinking, researchers use a series of gambits to try to see the world as it really is.”

## Data Availability

The original contributions presented in the study are included in the article/Supplementary material, further inquiries can be directed to the corresponding author.

## References

[B1] Bell A. C. West G. Felsenfeld G. (1999). The protein CTCF is required for the enhancer blocking activity of vertebrate insulators. Cell 98 (3), 387–396. 10.1016/s0092-8674(00)81967-4 10458613

[B2] Bird A. (2002). DNA methylation patterns and epigenetic memory. Genes Dev. 16, 6–21. 10.1101/gad.947102 11782440

[B3] Bird A. (2013). Dissolving the layers in genetics and epigenetics with Dr. Adrian Bird. Epigenie. Available at: http://epigenie.com/dissolving-the-layers-in-genetics-and-epigenetics-with-dr-adrian-bird (Accessed July 19, 2020).

[B4] Bird A. Taggart M. Frommer M. Miller O. J. Macleod D. (1985). A fraction of the mouse genome that is derived from islands of nonmethylated, CpG-rich DNA. Cell 40 (1), 91–99. 10.1016/0092-8674(85)90312-5 2981636

[B5] Bird A. P. (1980). DNA methylation and the frequency of CpG in animal DNA. Nucleic Acids Res. 8 (7), 1499–1504. 10.1093/nar/8.7.1499 PMC324012 6253938

[B6] Bird A. P. Southern E. M. (1978). Use of restriction enzymes to study eukaryotic DNA methylation: I. The methylation pattern in ribosomal DNA from *Xenopus laevis* . J. Mol. Biol. 118 (1), 27–47. 10.1016/0022-2836(78)90242-5 625056

[B7] Coulondre C. Miller J. H. Farabaugh P. J. Gilbert W. (1978). Molecular basis of base substitution hotspots in *Escherichia coli* . Nature 274, 775–780. 10.1038/274775a0 355893

[B8] Deaton A. M. Bird A. (2011). CpG islands and the regulation of transcription. Genes Dev. 25 (10), 1010–1022. 10.1101/gad.2037511 PMC3093116 21576262

[B9] Deichmann U. (2007). “Molecular” versus “colloidal”: controversies in biology and biochemistry, 1900–1940. Bull. Hist. Chem. 32 (2), 105–118.

[B10] Deichmann U. (2015). Chromatin: its history, current research, and the seminal researchers and their philosophy. Perspect. Biol. Med. 58 (2), 143–164. 10.1353/pbm.2015.0024 26750599

[B11] Deichmann U. (2016). Epigenetics: the origins and evolution of a fashionable topic. Dev. Biol. 416, 249–254. 10.1016/j.ydbio.2016.06.005 27291929

[B12] Evans T. Felsenfeld G. (1989). The erythroid-specific transcription factor eryf1: a new finger protein. Cell 58 (5), 877–885. 10.1016/0092-8674(89)90940-9 2776214

[B13] Feinberg A. P. Vogelstein B. (1983). Hypomethylation distinguishes genes of some human cancers from their normal counterparts. Nature 301 (5895), 89–92. 10.1038/301089a0 6185846

[B14] Felsenfeld G. (2014). The evolution of epigenetics. Perspect. Biol. Med. 57 (1), 132–148. 10.1353/pbm.2014.0004 25345707

[B15] Felsenfeld G. Davies D. R. Rich A. (1957). Formation of a three-stranded polynucleotide molecule. J. Am. Chem. Soc. 79 (8), 2023–2024. 10.1021/ja01565a074

[B16] Greally J. M. (2018). A user’s guide to the ambiguous word ‘epigenetics. Nat. Rev. Mol. Cell Biol. 19, 207–208. 10.1038/nrm.2017.135 29339796

[B17] Greger V. Passarge E. Höpping W. Messmer E. Horsthemke B. (1989). Epigenetic changes may contribute to the formation and spontaneous regression of retinoblastoma. Hum. Genet. 83 (2), 155–158. 10.1007/BF00286709 2550354

[B18] Henikoff S. Greally J. (2016). Epigenetics, cellular memory and gene regulation. Curr. Biol. 26, R644–R648. 10.1016/j.cub.2016.06.011 27458904

[B19] Holliday R. (1987). The inheritance of epigenetic defects. Science 238 (4824), 163–170. 10.1126/science.3310230 3310230

[B20] Mattei A. L. Bailly N. Meissner A. (2022). DNA methylation: a historical perspective. Trends Genet. 38, 676–707. 10.1016/j.tig.2022.03.010 35504755

[B21] Mohandas T. Sparkes R. S. Shapiro L. J. (1981). Reactivation of an inactive human X chromosome: evidence for X inactivation by DNA methylation. Science 211 (4480), 393–396. 10.1126/science.6164095 6164095

[B22] Morange M. (2002). The relations between genetics and epigenetics. A historical point of view. Ann. N. Y. Acad. Sci. 981, 50–60. 10.1111/j.1749-6632.2002.tb04911.x 12547673

[B23] Nanney D. L. (1958). Epigenetic control systems. Proc. Natl. Acad. Sci. 44, 712–717. 10.1073/pnas.44.7.712 PMC528649 16590265

[B24] Reik W. Collick A. Norris M. L. Barton S. C. Surani M. A. (1987). Genomic imprinting determines methylation of parental alleles in transgenic mice. Nature 328 (6127), 248–251. 10.1038/328248a0 3600805

[B25] Russo V. E. A. Martienssen R. A. Riggs A. D. (1996). “Introduction,” in Epigenetic mechanisms of gene regulation. Editors Russo D. H. Martienssen R. A. Riggs A. D. (New York, NY: Cold Spring Harbor Laboratory Press), 1–4.

[B26] Waddington C. H. (1942). The epigenotype. Endeavour 1, 18–20.

